# Mouse medulloblastoma driven by CRISPR activation of cellular *Myc*

**DOI:** 10.1038/s41598-018-24956-1

**Published:** 2018-06-07

**Authors:** BaoHan T. Vo, Jin Ah Kwon, Chunliang Li, David Finkelstein, Beisi Xu, Brent A. Orr, Charles J. Sherr, Martine F. Roussel

**Affiliations:** 1 Department of Tumor Cell Biology, St. Jude Children’s Research Hospital, 262 Danny Thomas Place, Memphis, TN 38105 USA; 2 Department of Computational Biology, St. Jude Children’s Research Hospital, 262 Danny Thomas Place, Memphis, TN 38105 USA; 3 Department of Pathology, St. Jude Children’s Research Hospital, 262 Danny Thomas Place, Memphis, TN 38105 USA; 4 Howard Hughes Medical Institute, St. Jude Children’s Research Hospital, 262 Danny Thomas Place, Memphis, TN 38105 USA

## Abstract

MYC-driven Group 3 (G3) medulloblastoma (MB) is the most aggressive of four molecular subgroups classified by transcriptome, genomic landscape and clinical outcomes. Mouse models that recapitulate human G3 MB all rely on retroviral vector-induced Myc expression driven by viral regulatory elements (Retro-Myc tumors). We used nuclease-deficient CRISPR/dCas9-based gene activation with combinatorial single guide RNAs (sgRNAs) to enforce transcription of endogenous *Myc* in *Trp53*-null neurospheres that were orthotopically transplanted into the brains of naïve animals. Three combined sgRNAs linked to dCas9-VP160 induced cellular *Myc* expression and large cell anaplastic MBs (CRISPR-Myc tumors) which recapitulated the molecular characteristics of mouse and human G3 MBs. The BET inhibitor JQ1 suppressed MYC expression in a human G3 MB cell line (HD-MB03) and CRISPR-Myc, but not in Retro-Myc MBs. This G3 MB mouse model in which Myc expression is regulated by its own promoter will facilitate pre-clinical studies with drugs that regulate *Myc* transcription.

## Introduction

Medulloblastoma (MB), the most common malignant pediatric brain tumor originating from the cerebellum, is classified into four major distinct molecular subgroups, including Wingless (WNT), Sonic Hedgehog (SHH), Group 3 (G3) and Group 4 (G4)^1,2^. Recently, similarity network fusion (SNF) applied to genome-wide DNA methylation, gene expression, somatic copy-number alterations, and clinical features of 763 primary samples further subdivided MBs into 12 different subtypes, with distinct characteristics with respect to age, gender, prognosis and response to therapy^3^. Regardless of the genetic, epigenetic and phenotypic differences of MB subgroups, patients generally receive a combination of surgery, radiation and chemotherapy^4^.

The G3 subgroup representing about 25% of all MBs is characterized by high MYC protein expression resulting from somatic *MYC* gene amplification in 15–20% of cases^5^. Large cell anaplastic G3 tumors with *MYC* amplification are associated with poor clinical outcome^5,6^. Several G3 mouse models have been developed by various methods including orthotopic transplantation of *Trp53*-null granule neural progenitors (GNPs) infected with retroviruses encoding Myc^7^ (hereafter referred as Retro-Myc); wild type GNPs infected with retroviruses expressing Myc and Gfi1^8^; wild type embryonic neural stem cells co-transduced with retrovirally expressed Myc and a dominant-negative (DN) form of Trp53^9^ or Gfi1^8,10^; or delivery of vectors expressing a conditional form of Myc and DN Trp53 into the embryonic cerebellum by *in utero* electroporation^7,9,11^. All these mouse models fully recapitulate human G3 MBs identified by cross-species gene expression analysis. However, they rely on the ectopic expression of *Myc* from a retrovirus long terminal repeat (LTR) or other constitutively active promoters in which Myc is no longer regulated by its endogenous transcriptional control elements. To date, only a handful of novel therapies for the treatment of G3 MB have been identified^12,13^. Therefore, generating mouse models of G3 MB which retain the physiological regulation of endogenous *Myc* is warranted for pre-clinical studies with drugs that suppress *Myc* transcription, such as bromodomain inhibitors (BETi)^14^.

CRISPR RNA and CRISPR-associated (Cas) proteins can generate RNA guided catalytic protein-RNA complexes to produce double-strand breaks at complementary DNA target sequences. Aspartic acid D10 and histidine H480 of the Cas9 nuclease from *Streptococcus pyogenes* are required for its nuclease activity^15,16^, enabling a catalytically defective Cas9 protein (dCas9) carrying alanine substitutions (D10A and H840A) to be employed in CRISPR gene targeting without cutting the genome^17^. dCas9 can be used in conjunction with fused effector domains such as VP16, p300, VPR or KRAB to epigenetically activate or suppress gene transcription^18–22^. To our knowledge, the application of dCas9 to enforce the expression of oncogenic drivers to induce tumor development *in vivo* has not been addressed. Here, we demonstrate the ability of the CRISPR-dCas9-VP160 system to modulate endogenous *Myc* expression in *Trp53*-null neurosphere cells to generate an orthotopic mouse model of G3 MB amenable to BETi treatment.

## Results

### Design of CRISPR activation of endogenous *Myc*

**Figure 1:**
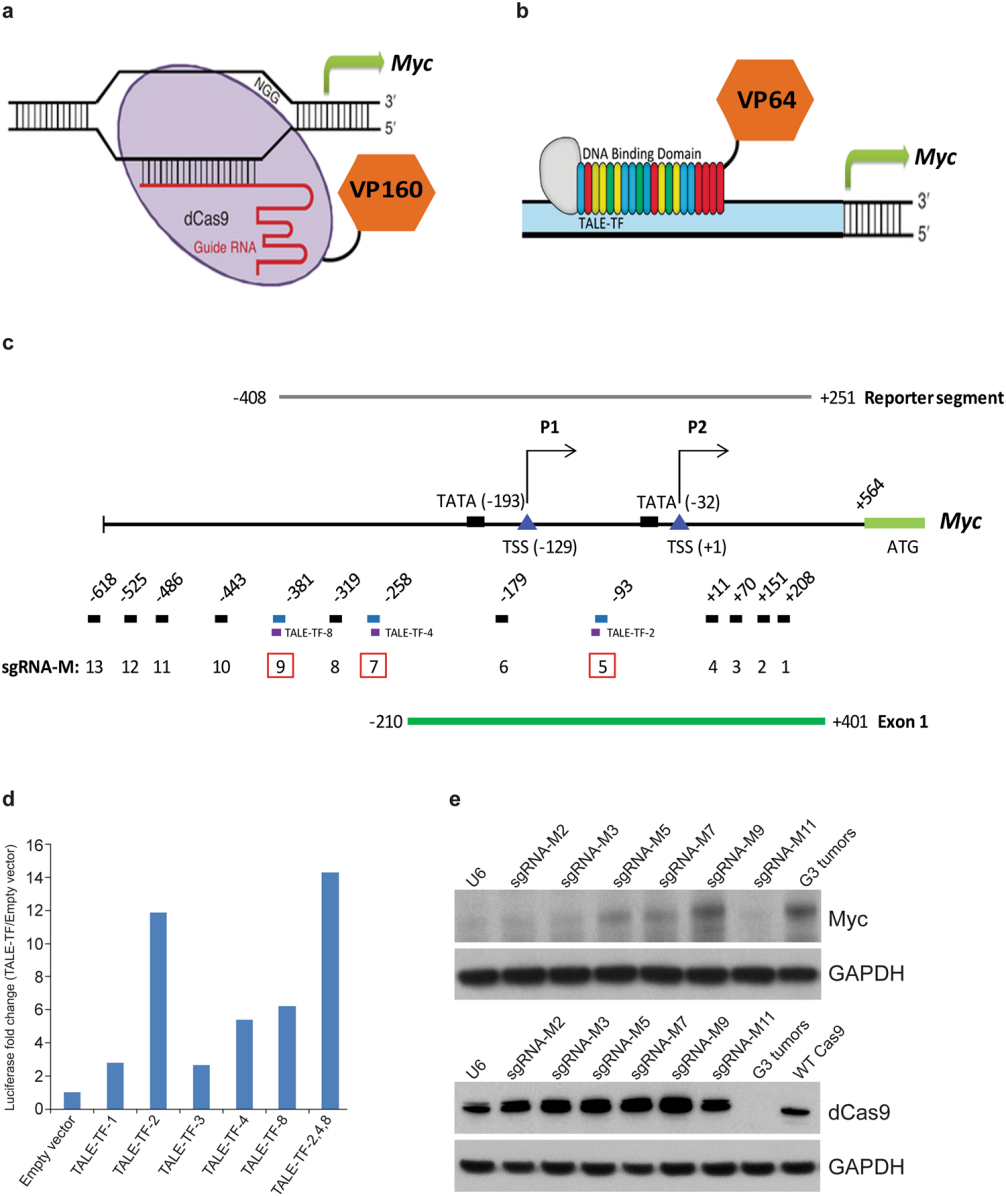
Design of CRISPR activation of endogenous Myc. Schematic diagram of (**a**) CRISPR and (**b**) TALE-TF activation. Nuclease-deficient dCas9 or TALE 20-mers were fused to VP16 with tandem repeats as VP64 or VP160. (**c**) Schematic diagram of the mouse *Myc* promoter and genome editing designs to activate the expression of endogenous *Myc* by TALE-TF and CRISPR (sgRNA-M). Red boxes highlight the overlapping loci targeted by both genome editing approaches. (**d**) Co-transfection of the *Myc* promoter-driven luciferase reporter plasmid with different TALE-TF constructs targeting the *Myc* promoter into NIH3T3 cells, followed by luciferase assay to quantify luciferase levels as proxy to *Myc* activation. (**e**) NIH3T3 cells were infected with Lenti-dCas9-VP64-T2A-GFP and several individual sgRNAs targeting the *Myc* promoter. Cell lysates were harvested for immunoblotting using antibodies against Myc, dCas9, and GAPDH used as loading control. A mouse G3 MB line (#19251/MB1) was used to document Myc levels. Full blots are presented in Supplementary Fig. S4.

Using previous H3K27acetylation chromatin immunoprecipitation sequencing (ChIP-Seq) and ATAC-Seq data from purified mouse G3 Retro-Myc MBs^8^, we located a ~1.2 Kb open chromatin region corresponding to the cellular *Myc* dual P1 and P2 promoter region (Supplementary Fig. S1) to which we designed a series of CRISPR guide RNAs. To facilitate gene activation, we fused sequences encoding 4X or 10X tandem repeats of the transactivation domain of *Herpes simplex* virus protein VP16 (VP64 or VP160, respectively) to the C-terminus of nuclease-deficient dCas9 (D10A, H840A) and fused these to T2A-GFP in a lentivirus backbone or transposon vector^23^ (Fig. 1a). Alternatively, we used sequences encoding a group of transcription activator-like effector (TALE) polypeptides fused to VP64 and T2A-GFP^24^ (Fig. 1b). CRISPR and TALE design software^8,25^ pinpointed 13 sgRNAs (sgRNA-M1 to M13) and 8 TALE binding motifs (TALE-TF-1 to -8) within a ~1.2 Kb segment upstream of the initiator ATG of the cellular *Myc* gene. These sgRNA and TALE sequences were compared against the whole mouse genome using the NCBI BLAST nucleotide program to rule out adventitiously targeted loci. Both design strategies recognized three overlapping target loci designated sgRNA-M5, -M7, and -M9 and TALE-TF-2, -4 and -8 (Fig. 1c).

The retro-Myc MB model was generated by enforced high titer vector-mediated-Myc expression in *Trp53*^−/−^; *Cdkn2c*^−/−^ GNPs which were orthotopically transplanted into the cortices of recipient nude mice, as previously described^7^. Retro-Myc MBs are polyclonal tumors containing multiple, randomly integrated proviral DNA sequences that, in aggregate, encode high levels of Myc RNA and protein^8^. To identify TALE and sgRNAs that activate cellular *Myc* expression to levels approaching those found in mouse G3 Retro-Myc MBs, we cloned a 0.665 Kb minimal promoter that includes two TATA box sequences and TALE-TF binding motifs upstream of the reporter *Luciferase* gene in a pGL3 expression vector^26^. NIH3T3 cells were co-transfected with or without *Myc* promoter-reporter plasmids and TALE-TF vectors. Compared to the empty vector, TALE-TFs-1, 2, 3, 4 and 8 upregulated reporter activity more than 2-fold with TALE-TFs-2, 4 and 8 being most effective (Fig. 1d). Combined use of TALE-TFs -2, -4 and -8 induced luciferase activity 14-fold (Fig. 1d). In turn, NIH3T3 cells infected with a lentivirus vector encoding both dCas9-VP64 and GFP were used to generate stable cell lines that were subsequently infected with lentiviruses expressing U6 promoter-driven sgRNAs co-expressing RFP. Immunoblotting of double-positive GFP^+^/RFP^+^ cells selected by fluorescence-activated cell sorting (FACS) with antibodies to Myc and dCas9 showed that sgRNA-M5, -M7 and -M9, closely corresponding to TALE-TF-2, -4 and -8, induced endogenous *Myc* expression (Fig. 1e). Myc levels induced by sgRNA-M9 mimicked those achieved in G3 tumors from Retro-Myc MB cells (Fig. 1e). No activation was detected with sgRNA-M2, M3 or M11. Because three similar sequences in the *Myc* promoter targeted by both TALE-TF and sgRNAs led to significant Myc expression, subsequent experiments were continued using CRISPR alone.

### Activation of cellular *Myc* in *Trp53*-null GNPs generates G3 MBs

**Figure 2:**
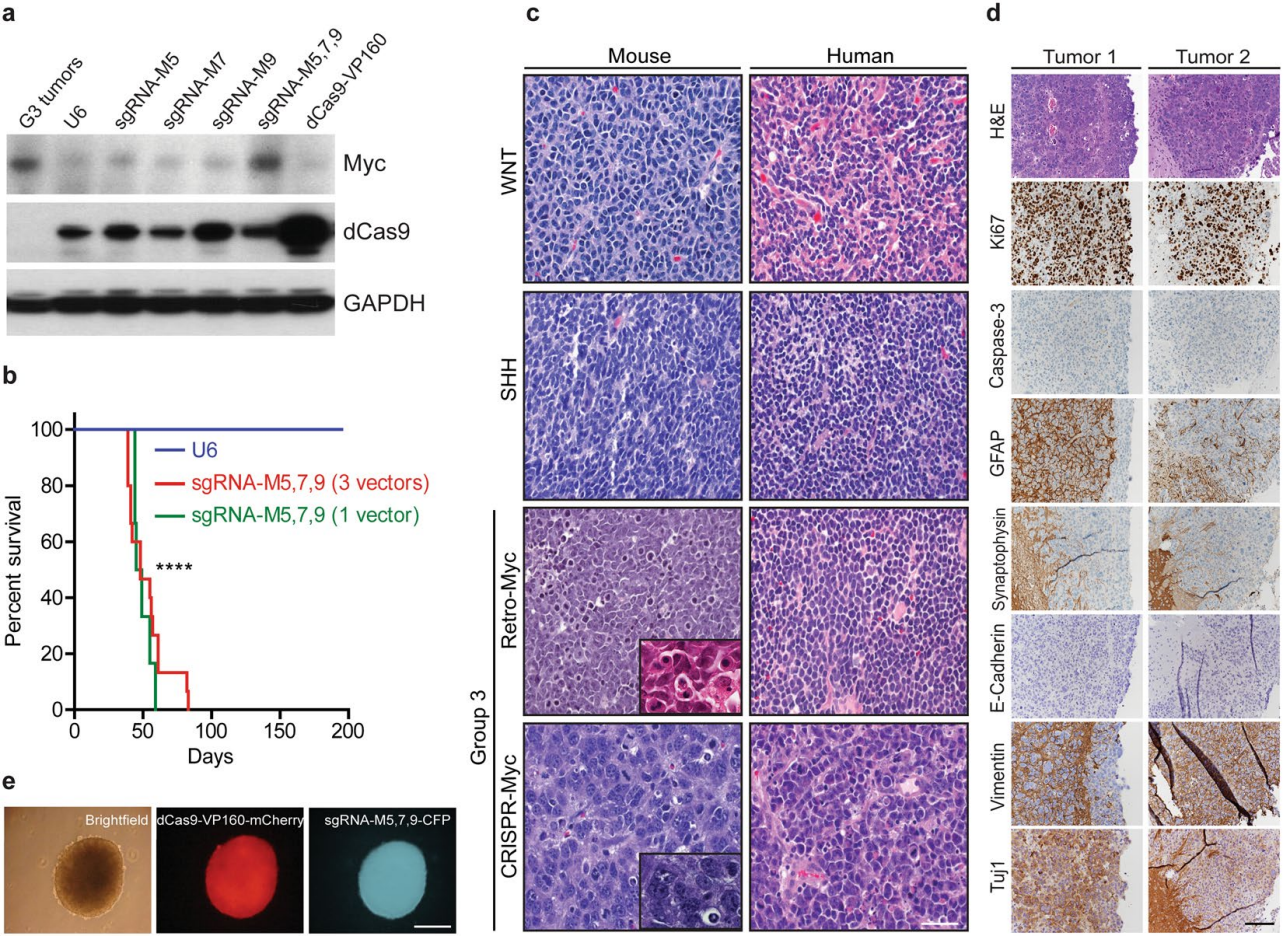
Characterization of Myc activation in CRISPR-Myc MBs. (**a**) Detection of Myc and dCas9 protein by immunoblotting of *Trp53*-null neurosphere cells stably expressing pT2K-CAGGS-dCas9-VP160-T2A-Puro-mCherry and sgRNA-M5, M7, M9 as single guides and as a pool sgRNA-M5,7,9. G3 tumors were used as positive control for Myc levels. GAPDH was used as loading control. Full blots are presented in Supplementary Fig. S5. (**b**) Kaplan-Meier survival curves for mice orthotopically transplanted with engineered *Trp53*-null neurospheres stably expressing pT2K-CAGGS-dCas9-VP160-T2A-Puro-mCherry and the pool expressing sgRNA-M5, M7 and M9 (n = 15) or a single vector construct expressing all three guides sgRNA-M5,7,9 (n = 6). Median survival time for mice bearing tumors: 48 days post-transplantation. Negative control cells expressed pT2K-CAGGS-dCas9-VP160-T2A-Puro-mCherry without sgRNAs (n = 5). ****p < 0.0001. (**c**) Hematoxylin and Eosin (H&E) staining of mouse and human WNT, SHH, G3 (Retro-Myc), and G3 (CRISPR-Myc) MBs showing large cell anaplastic features typify G3 MBs as shown in inserts. Scale bar, 100 µm. (**d**) Sections of CRISPR-Myc tumors immunostained with antibodies to H&E, Ki67, Caspase-3, GFAP, Synaptophysin, E-Cadherin, Vimentin, and Tuj1. Scale bar, 200 µm. **(e**) Tumors harvested from (b) cultured as neurospheres showed stable expression of dCas9-VP160 (mCherry) and sgRNA-M5, 7, 9 (CFP). Scale bar, 100 µm.

To determine if cells expressing trans-activated *Myc* generated MBs, we transiently transfected *Trp53*-null GNPs grown as neurospheres with a pT2K Tol2 transposon vector^11^ co-encoding dCas9-VP160 and mCherry together with a non-integrating vector encoding the Tol2 transposase (T2TP). Neurospheres were co-transfected with a third pT2K vector specifying cyan fluorescent protein (CFP) and sgRNA-M5, -M7 and -M9, either individually or in combination. Pooled mCherry and CFP double-positive neurospheres were isolated by FACS. Individual sgRNA-M5, -M7, and -M9 guides induced *Myc* RNA expression (Supplementary Fig. 2a and b), while the combination of the three sgRNAs increased Myc protein to levels similar to those in a mouse G3 MB Retro-Myc tumor (Fig. 2a). Hence, we cloned sgRNA-M5, -M7 and -M9 into the U6-guide RNA cassette, and co-expressed these guides in a single vector backbone. FACS sorted mCherry^+^/CFP^+^ cells (total 1 × 10^6^ per mouse) expressing dCas9-VP160 and sgRNA-M5, -M7 and -M9 using either three independent vectors or one vector were orthotopically transplanted into the cerebral cortices of 6 to 8-week-old recipient nude mice^8^. As a negative control, an equal number of cells expressing dCas9-VP160 without sgRNAs were implanted. All mice implanted with *Trp53*-null neurospheres expressing dCas9-VP160 with the combination of three sgRNA-M5, -M7 and -M9 developed (CRISPR-Myc) tumors, whereas none were generated in the negative control group during 6-months of observation (Fig. 2b). In several cases, tumors metastasized to the spinal cord, consistent with the aggressive nature of Myc-driven G3 MBs. The presence of all three sgRNAs in the tumors was confirmed by genomic PCR and nucleotide sequencing. Therefore, the co-recruitment of dCas9-VP160 to sgRNA-M5, -M7 and -M9 binding sites was sufficient to induce levels of endogenous Myc expression compatible with tumor development *in vivo*.

**Figure 3:**
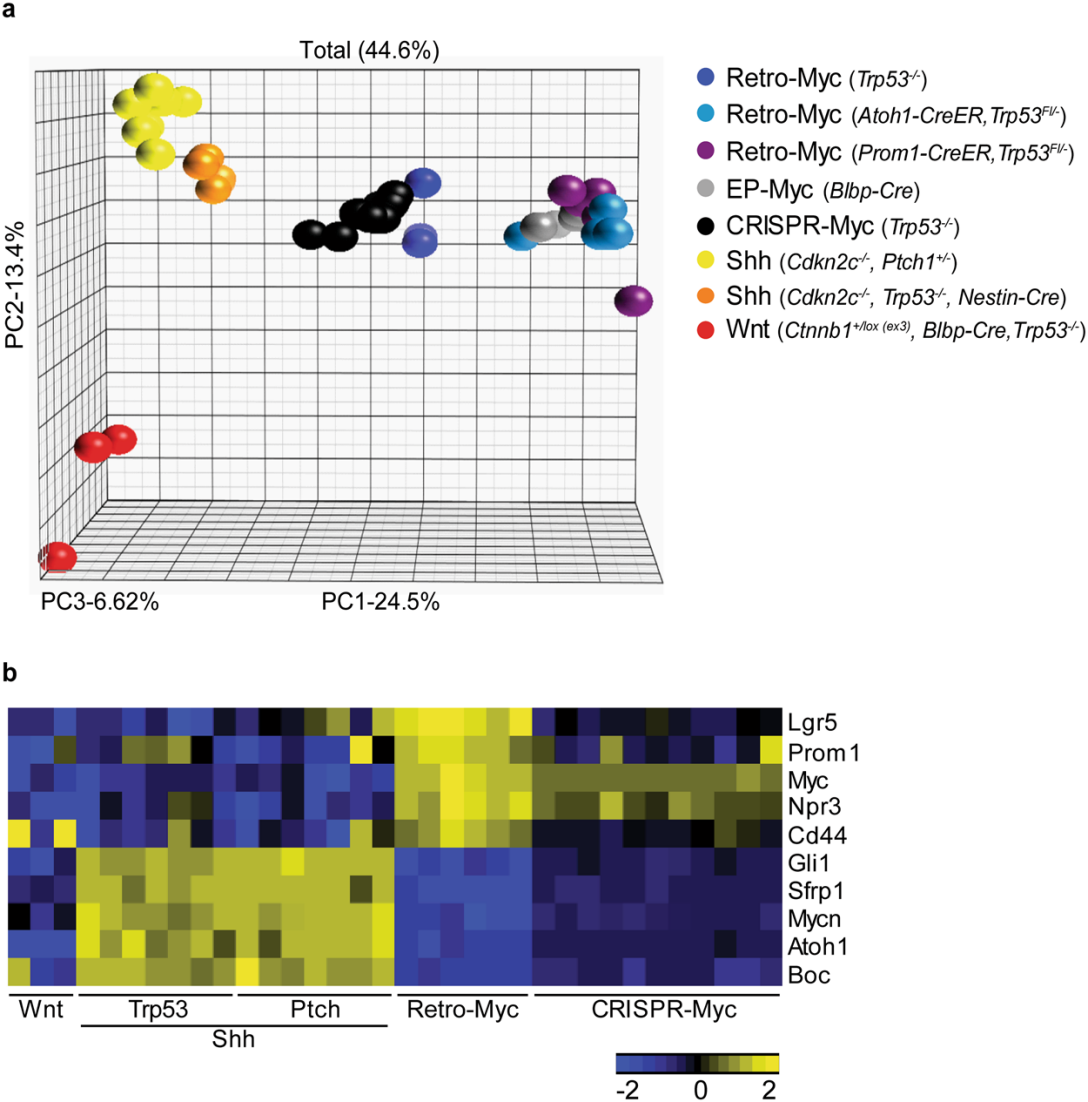
CRISPR induced Myc-driven MBs molecularly recapitulate other mouse G3 MB models. (**a**) Principal component analysis across mouse MB models from the Affymetrix gene chip microarray. Retro-Myc: orthotopically derived tumors from infection of (*Trp53*^−/−^, n = 3), (*Atoh1-CreER, Trp53*^−/−^, n = 4) and (*Prom1-CreER, Trp53*^−/−^, n = 5) purified GNPs with retroviruses expressing *Myc*. EP-Myc: *in utero* electroporation (EP) of Myc and T2TP with DN p53 transposon system in *Blbp-Cre* (n = 4) embryos at embryonic day 13.5. CRISPR-Myc (*Trp53*^−/−^): dCas9-VP160 (n = 11) in combination with specific sgRNAs to activate endogenous Myc expression in *Trp53*-null GNPs. *Ptch1* or *Trp53*-Shh: spontaneously arising tumors from (*Cdkn2c*^−/−^, *Ptch1*^+/−^, n = 8) and (*Cdkn2c*^−/−^*, Trp53*^*fl/fl*^*, Nestin-Cre*, n = 5) mice, respectively. Wnt: mouse model of Wnt tumors from (*Ctnnb1*^+/*lox(ex3)*^*, Blbp-Cre, Trp53*^−/−^, n = 3) mice. (**b**) Heat map of differentially regulated genes between the Shh-subgroup from *Ptch1*^+/−^ and *Trp53*-null mice, Retro-Myc, CRISPR-Myc, and Wnt-subgroup tumors.

When compared to MBs arising in mouse and human WNT, SHH and two Retro-Myc G3 MB mouse models, the CRISPR-Myc tumors shared cardinal properties with Retro-Myc G3 tumors, including large cell anaplastic features (Fig. 2c) and canonical histological markers (Fig. 2d). *In vitro*, tumor cells grew as neurospheres, could be expanded for multiple passages, and stably expressed the mCherry and CFP fluorescence markers that were fused to the dCas9-VP160 and sgRNA vectors (Fig. 2e). We performed microarray analyses and compared the CRISPR-Myc tumors to other previously derived mouse G3 MBs initiated through independent approaches^7,11^, as well as to Shh and Wnt^27^ mouse MBs^27,28^. Principal component analysis (PCA) revealed that CRISPR-Myc tumors clustered together and were most similar to Retro-Myc MBs, while clearly different from Shh and Wnt tumors (Fig. 3a). As expected, a subset of genes that typify three subgroups of MB were differentially expressed among individual subgroups (Fig. 3b). Elevated *Myc* expression itself, higher in Retro-Myc than in CRISPR-Myc MBs, as well as that of *Npr3*, a hallmark of human G3 MBs, were restricted to both mouse G3 Myc MB models. Conversely, lower levels of Shh-associated genes, *Mycn*, *Gli1*, *Atoh1*, *Sfrp1* and *Boc*, were detected in Retro- and CRISPR-Myc MBs. However, *Cd44* levels were higher in Retro-Myc MBs than in CRISPR-Myc MBs, as were those of *Prom1* and *Lgr5*, two markers of stem-like cells (Fig. 3b). Although the latter differences in gene expression between the two Myc MB models correlate with higher overall levels of viral versus cellular *Myc* expression induced in these tumors, the levels of CRISPR-Myc were sufficient to reproducibly yield G3 MBs.

### Validation of sgRNAs specific binding to the *Myc* promoter upon CRISPR activation

**Figure 4:**
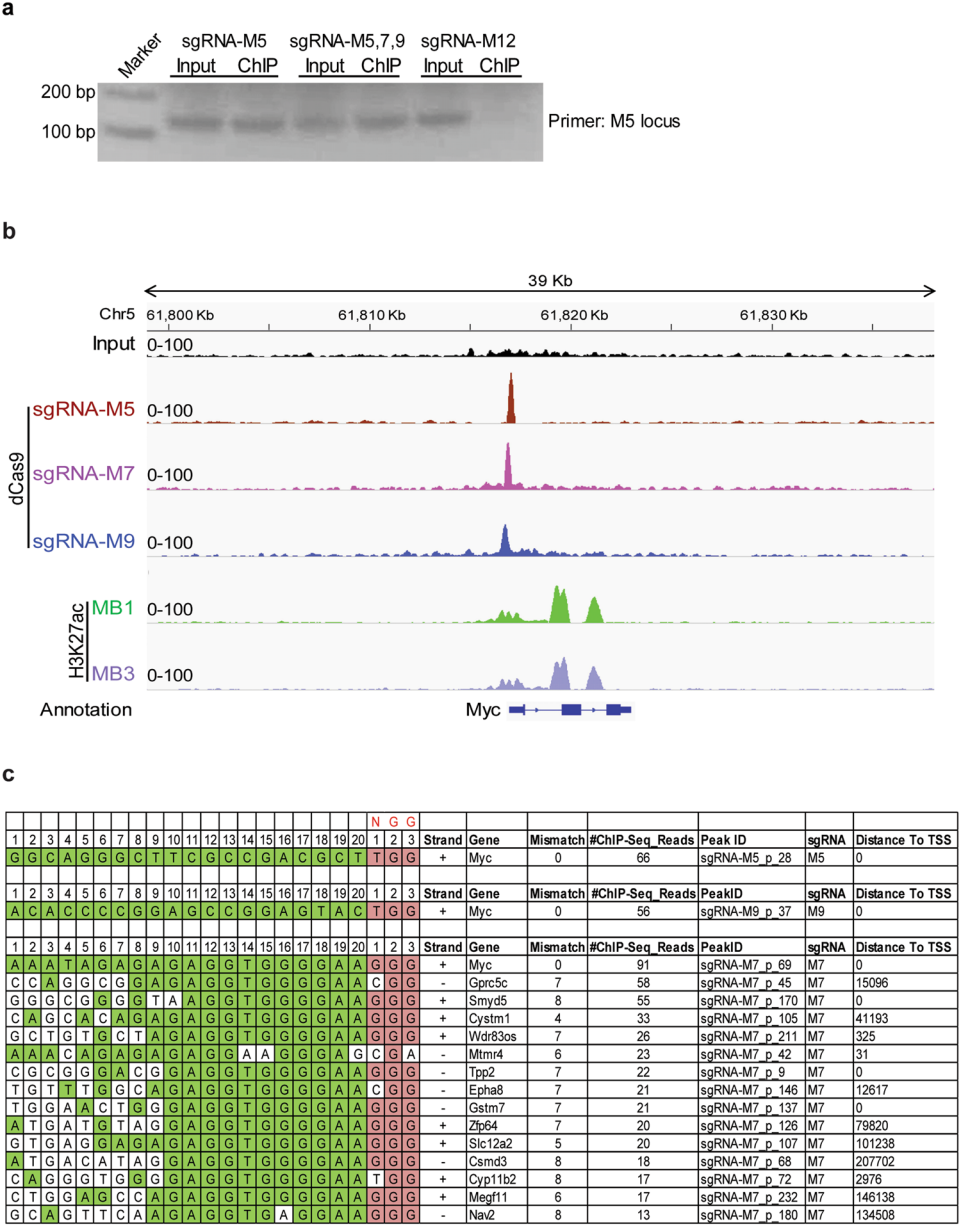
Validation of sgRNAs specific binding to the endogenous Myc promoter in CRISPR activation system. (**a**) Primers flanking the sgRNA-M5 binding motif was used for ChIP-PCR in *Trp53*-null neurosphere cells infected with Lenti-dCas9-VP64-T2A-GFP and Lenti-U6-sgRNA-RFP with sgRNA-M5 or combination with sgRNA-M5, 7, and 9. sgRNA-M12 was used as a negative control. Full agarose gel is presented in Supplementary Fig. S6. (**b**) Enrichment of sgRNA-M5, M7 and M9 binding to pre-designated loci in *Trp53*-null neurosphere cells revealed by ChIP-Seq using dCas9 antibody. ChIP-Seq of H3K27ac on mouse G3 MB cell lines MB1 and MB3 illustrated to define open chromatin status of the endogenous Myc locus. (**c**) Mapping reads from ChIP-Seq of dCas9 used to characterize on-target (M5, M9) and off-target (M7) sequences.

To validate the binding specificity of dCas9 to the pre-designed segments directed by sgRNAs, we performed ChIP, as previously described^29^, using an antibody against dCas9 for analysis of *Trp53*-null neurospheres expressing dCas9-VP64 and sgRNA-M5, -M7 and -M9. PCR analysis of the ChIP showed substantial enrichment of dCas9 protein at the M5 genomic segment (Fig. 4a). In agreement, ChIP-Seq peak analysis clearly demonstrated dCas9 binding at each of the three intended target sites (Fig. 4b). By analyzing peaks across the whole genome, no binding sites other than the on-target sequences were detected with sgRNA-M5 and -M9. However, for sgRNA-M7, although most reads were on-target, we resolved a number of off-target binding sites with near perfect alignment to proximal protospacer adjacent motifs (PAMs) but with distal mismatches (Fig. 4c). Off-target binding sites resided on non-coding intronic regions of other genes (e.g. *Gprc5c* and *Cystm1*) except for the promoter of *Smyd5*, a histone methyltransferase for H4K20 in heterochromatic regions that maintain embryonic stem (ES) cell lineage specification^30^ (Supplementary Fig. 3).

### BET bromodomain inhibitor JQ1 suppresses MYC expression in human and mouse MB cells

**Figure 5:**
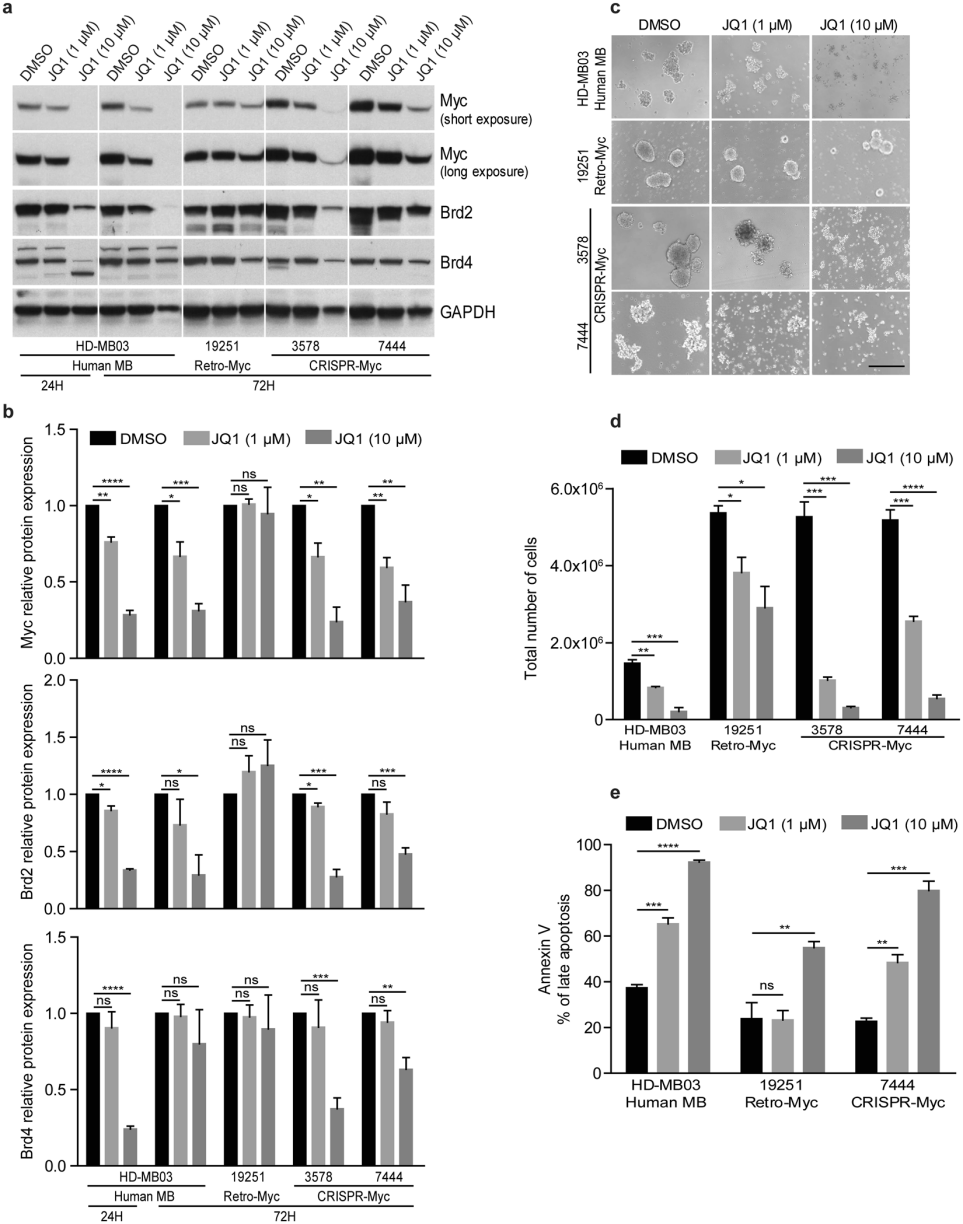
BET inhibitor JQ1 suppresses Myc protein expression in human and mouse G3 MB cell lines. (**a**) G3 MB mouse cell lines Retro-Myc (#19251) and CRISPR-Myc (#3578 and #7444) and human HD-MB03 cells were treated with JQ1 at the indicated concentrations and times. Immunoblotting detected Myc, Brd2, and Brd4 protein expression levels. GAPDH was used as loading control. Full blots are presented in Supplementary Fig. S7. (**b**) Quantitative analysis of Myc, Brd2, and Brd4 protein levels in human and mouse cells treated with JQ1 relative to the untreated control (designated as 1.0) after normalization to the signal obtained with GAPDH. Data are represented as the mean ± SEM (n = 3). *p = 0.0265, p = 0.0234, p = 0.0245, p = 0.0193, and p = 0.0159; **p = 0.0017, p = 0.0013, p = 0.0090, p = 0.0045, and p = 0.0031; ***p = 0.0003; p = 0.0006, and p = 0.0009; ****p < 0.0001; ns, not significant. (**c**) Representative images of human and mouse MB tumorsphere cells treated with JQ1 at 1 and 10 μM for 72 hours. Scale bar, 100 µm. (**d**) Total number of tumorsphere cells were counted after 72 hours JQ1 treatment. Data are represented as the mean ± SEM (n = 3). *p = 0.0138 and p = 0.0238; **p = 0.0029; ***p = 0.0002, p = 0.0004, and p = 0.0008; ****p < 0.0001. (**e**) Percentage of apoptotic cells from human and mouse MB cell lines after treatment with 1 and 10 μM JQ1 for 72 hrs. Data are represented as the mean ± SEM (n = 3). *p = 0.0007; **p = 0.0030 and p = 0.0098; ***p = 0.0002; ****p < 0.0001; ns, not significant.

Brd4, a member of the bromodomain and extra-terminal (BET) protein family that reads acetylated lysines was previously shown to activate *MYC* transcription in acute myeloid leukemia (AML)^31^. Conversely the small molecule Brd4 inhibitor JQ1 effectively inhibited MYC expression in this leukemia but was unable to do so in models in which MYC was ectopically expressed^31^. A previous study in a G3 MB line (MB002) showed that JQ1 suppresses stem cell signaling and inhibits tumor cell self-renewal and development^32,33^. The human (HD-MB03) and mouse Retro-Myc (#19251) and CRISPR-Myc (#3578 and #7444) MB tumor cell lines were treated with 1 and 10 μM of JQ1 for 24 or 72 hours. Treatment with 10 μM of JQ1 for 72 hrs inhibited MYC, BRD2, and BRD4 protein expression levels in human and CRISPR-Myc MB cell lines without affecting expression of these proteins in the Retro-Myc MB cell line (Fig. 5a,b). In turn, tumorsphere formation of human MB and CRISPR-Myc versus Retro-Myc MBs was inhibited (Fig. 5c,d) and associated with increased apoptosis (Fig. 5e).

## Discussion

Among the known molecularly characterized MB subgroups, the highest levels of MYC expression and gene amplification typify aggressive G3 tumors^34,35^. Mouse models of MB that recapitulate the cardinal features of human G3 tumors were previously generated in *Trp53*-deficient GNPs by retroviral gene transfer^7,9^ in which insertion of multiple *Myc*-encoding proviruses induced polyclonal populations of tumor cells that in aggregate expressed sufficiently high levels of Myc protein to yield G3 tumors^8^. The major limitations of past models have been that retrovirally-driven gene expression bypasses normal regulation of *Myc* by its endogenous regulatory elements; that tumor latency in individual experiments is highly variable; and that the contribution of proviral insertional mutagenesis is difficult to exclude. By using the CRISPR activation system^23^ to induce endogenous *Myc* expression in *Trp53*-null GNPs, we have now generated aggressive large cell anaplastic MBs driven by the cellular *Myc* gene that exhibit the canonical histological and molecular properties of the G3 subgroup.

Optimization of CRISPR-driven gene activation was previously undertaken by fusing dCas9 with different effectors, including concatemerized VP16 and p300 core domains^19,23,36^. Most of these models utilize sgRNAs targeting regions proximal to the transcriptional starting site (TSS), while the p300 core domain also works on distal enhancers^37^. By systematically comparing a series of more than 20 effectors with known transcriptional activity, the hybrid of VP64-p65-Rta (VPR) was found to exhibit the most potency for activating endogenous genes^38^ and was applied to promote the differentiation of human induced pluripotent stem (iPS) cells to neurons (iNeurons) by targeting Neurogenin2 (*NGN2*) or the Neurogenic Differentiation Factor 1 (*NEUROD1*)^38^. Alternatively, gene activation by protein-based TALE-TFs targeting the *Oct4* distal enhancer in conjunction with enforced expression of *Myc*, *Klf4*, and *Sox2* reprogrammed mouse embryonic fibroblast (MEF) cells into iPS cells in 3–4 weeks^39^. Following on such studies, we successfully piloted the use of both CRISPR/dCas9-VP160 and TALE-VP64 in activating cellular *Myc* and found that three particular and similar segments within the *Myc* promoter facilitated its induction by either method. We exploited CRISPR/dCas9 for subsequent experiments, acknowledging the greater convenience of the CRISPR system particularly in broader applications such as unbiased large-scale genome-wide screening^23,40^, regardless of the models used or phenotypic outputs.

Although the CRISPR system has enabled highly efficient genome editing in a wide variety of organisms^41^, unwanted off-target effects may present problems. Several studies have used high-fidelity Cas9 (SpCas9-HF)^42^, mutant scaffold RNA structures^43^ and truncated sgRNA with complementarity lengths of 17 or 18 nucleotides other than 20 nucleotides^44^, to avoid such complications. In our study, we used genome-wide ChIP-Seq to identify potential off-target binding sites. For sgRNA-M5 and M9, we did not detect any off-target binding sites. However, for sgRNA-M7, we identified 10 off-target lower affinity binding sites, many in introns, compared to on-target controls. Open chromatin from ATAC-Seq analysis showed that not all off-target binding sites had H3K27ac marks with the exception of *Smyd5*, implying that transcriptional activation need not follow from sgRNA binding.

Recent analysis using a larger cohort of patients subdivided MBs into multiple specific subtypes within each of the four major MB subgroups according to clinical outcome, gene methylation, transcriptome and genomic landscapes^3,45,46^. Many more potential driver genes were identified within these specific subgroups which might be validated using the CRISPR activation system as described here. A potentially interesting target in G3 MB is *Gfi1*, the activation of which in conjunction with *Myc* bypasses the requirement for *Trp53* loss of function in generating MBs^8,10^. However, our preliminary attempts to target this gene have not as yet yielded useful information. Unlike Retro-Myc tumors, the CRISPR-Myc MBs retain a heightened sensitivity to the BRD4 inhibitor JQ1, providing proof of principle that this new model might also provide a substrate for pre-clinical identification of small, potentially therapeutic molecules that regulate *Myc* transcription.

## Methods

### Animal husbandry

CD-1 *nu/nu* mice (Charles River Laboratories) were used as recipients for cortical transplants. *Trp53*-null mice in a C57BL/6 background were utilized to generate GNPs, as previously described^7,8^. All animal experiments were approved by, and conducted in accordance with St. Jude Children’s Research Hospital Animal Care and Use Committee guidelines, as required by the United States Animal Welfare Act and the National Institutes of Health’s policy to ensure proper care and use of laboratory animals for research.

### Vector construction

The lenti-dCas9-VP64-T2A-GFP plasmid was purchased from Addgene (#61422). The dCas9 contains two point mutations: D10A and H840A, which disrupt the nuclease activity of Cas9. We generated the sgRNA cloning vector Lenti-U6-sgRNAs-RFP by inserting a U6-sgRNA cassette into pCDH-CMV-MCS-EF1-RFP backbone (System Biosciences, CD512B-1) after cleavage by *SnaBI* and *EcoRI*. All target specific sgRNAs were predicted by online software (http://crispr.mit.edu/). Oligomers containing 20 bp of selected target sequence (Supplementary Table S1) were cloned into the sgRNA cloning vector at *BsmBI* sites. To generate TALE-TF-VP64 plasmids targeting different segments of the Myc promoter, sequences of TALEs predicted by online software (http://crispr.mit.edu/) were compared against the whole mouse genome using the NCBI nucleotide BLAST program to rule out alternative binding loci. TALE monomers were assembled using a commercial tool kit (Addgene, #000000019) as described previously^25^. Eighteen assembled monomers were inserted into one of four backbone plasmids, each of which included an N-terminal signal specific for thymidine and one of four base-specific C-terminal TALEs to generate a 20-nucleotide recognition sequence. These were fused in frame at the N-terminus with three Flag tags and a nuclear localization sequence, and at the C-terminus to sequences encoding the trans-activator module VP64. Correctly assembled TALEs cDNAs were verified by nucleotide sequencing analysis; transfection of cDNAs into human 293 T cells using Lipofectamine 2000, followed by immunoblotting of cell lysates with antibodies to the FLAG epitope (M2; Sigma-Aldrich), confirmed the expression of proteins of the expected masses (~135 kDa).

pT2K-CAGGS-dCas9-VP160-T2A-Puro-IRES-mCherry and pT2K-U6-stuffer-CAGGS-IRES-CFP were made by Gibson Assembly as described previously^47^. Briefly, the pT2K-CAGGS-IRES-CFP backbone was digested with *SalI* followed by gel extraction. The U6 stuffer insert was amplified from Lenti-V6 using U6 Forward primer 5′-AATAATCAATGTCGA GAGGGCCTATTTCCCATGATTCCT-3′ and U6 Reverse primer 5′-ATGGGCCCTCGTCGACCAATTCCCACTCCTTTCAAGACC-3′. dCas9-VP160-T2A-Puro was released from pAC94-pmax-dCas9-VP160-T2A-Puro by *EcoRI* digestion and cloned into EcoRI digested pT2K-CaGGS-IRES-mCherry by Gibson Assembly^47^. sgRNA-M5, -M7, and -M 9 were cloned individually into pT2K-U6-stuffer-CAGGS-IRES-CFP. pT2K-U6-stuffer-CAGGS-IRES-CFP was digested with *BsmBI* to remove the stuffer DNA and annealed guide RNAs were inserted using T4 ligation (Clontech, #6023).

pT2K-U6-sgRNA-M5-CAGGS-IRES-CFP was used as the backbone to clone pT2K-U6-sgRNA-M5, 7, 9-CAGGS-IRES-CFP. pT2K-U6-sgRNA-M7-CAGGS-IRES-CFP was used as a template to amplify the sgRNA-M7 along with the U6 promoter and sgRNA scaffold using 5′-ATAGGCCCTCCCAATTCCCACTCCTTTCAAGAC-3′ and 5′-AAGGAGTGGGAATTGGTC GAGAGGGCCTATTTCCCA-3′. pT2K-U6-sgRNA-M9-CAGGS-IRES-CFP was used as a template to amplify the sgRNA-M9 along with the U6 promoter and sgRNA scaffold using 5′-GCCAGATGGGCCCTCGTCGACCAATTCCCACT-3′ and 5′-TGGGAATTGGGAGGGCCT ATTTCCCATGATTCCT3′. The amplified inserts for sgRNA-M7 and M9 (U6 sgRNA scaffold) were cloned by Gibson Assembly^47^ into pT2K-U6-M5-CAGGS-IRES-CFP digested with *SalI*.

### Luciferase reporter assay

The mouse minimal *Myc* promoter that includes a 0.665 Kb fragment upstream of the initiating ATG codon was cloned into the pGL3 vector (Promega). For transfection, 1 × 10^5^ NIH3T3 cells were plated into 12-well plates overnight. A mixture of 0.5 μg of Myc reporter and 1 μg of different TALE-TF-VP64 plasmids were used for each transfection as described previously^8^. Two days after transfection, luciferase reporter assays were carried out following the manufacturer’s instructions (Promega).

### Cell culture, transfection, protein analysis, cell count and Annexin-V staining

Mouse NIH3T3, primary MEFs, ES cells, and GNPs purified from cerebella of 7-day-old *Trp53*-null mice, derivation of the mouse Retro-Myc tumor cell line (#19251) and CRISPR-Myc tumor cell lines (#3578 and #7444), and culture of human HD-MB03 MB cells were performed as described previously^8,25,48^. For GNPs and NIH3T3 cell transfection, 1 × 10^6^ cells were transfected with 2.5 μg of each TALE-TF-GFP or pT2K-CAGGS-dCas9-VP160-T2A-Puro-mCherry and pT2K-CAGGS-T2TP and pT2K-U6-sgRNAs-CAGGS-IRES-CFP carrying sgRNA-M5, M7 and M9 individually and in combination using Xfect Transfection Reagent (Clontech). Cell pellets were lysed in RIPA buffer, and proteins separated on denaturing gels were immunoblotted with primary antibodies^7^ against Myc (1:1000, #9402, Cell Signaling Technology), Brd2 (1:1000, #5848, Cell Signaling Technology), Brd4 (1:1000, #128874, Abcam), dCas9 (1:5000, 632607, Clontech), and GAPDH (1:5000, #AM4300, Applied Biosystems, Ambion).

Apoptosis was detected by flow cytometry using Annexin-V-APC according to the manufacturer’s protocol (Pharmingen, #550475). To assess cell number, MB tumorsphere cells were dissociated with accutase (A-11105-01, Invitrogen, Carlsbad, CA) and counted using 0.4% Trypan blue stain (Gibco by Life Technologies) in the countess automated cell counter (Invitrogen). Briefly, 3 × 10^5^ MB tumorsphere cells were plated in 6 well plates followed by treatment with 1 µM or 10 µM of JQ1 for 72 hours before cell count, Annexin-V and propidium iodide (PI) staining.

### Histopathology and immunohistochemistry

For histopathology, samples were formalin-fixed, paraffin-embedded, and sectioned at 5 μm thickness. For each sample, a section was stained with hematoxylin and eosin. For immunohistochemistry, tumor sections were subjected to heat antigen retrieval with 10 mM citrate buffer for 30 minutes. Sections were blocked for 30 minutes in 10% normal goat serum in phosphate buffered saline (PBS). Sections were incubated with specific antibodies: Ki67 (1:1000, #556003, BD Pharmingen), GFAP (1:200, Chemicon), Synaptophysin (1:100, #RM-9111-S, Thermo Fisher Scientific, Rockland, IL) and cleaved Caspase-3 (1:100, #CP229C, BioCare Medical, Concord, CA). Sections were then incubated for 60 minutes with biotinylated goat anti-rabbit IgG (Vector labs) at 1:200 dilutions. Blocker D, Streptavidin- HRP and AEC detection kit (Abcam) were used according to the manufacturer’s instructions. Representative images of each sample/stain combination were captured and analyzed using Axiovision software (Carl Zeiss Microscopy).

### Lentivirus infection and orthotopic transplantation

High titer lentivirus stocks were generated in 293 T cells as previously described^7^. GNP-derived neurospheres were infected with Lenti-dCas9-VP64-T2A-GFP and Lenti-U6-sgRNAs-RFP, sorted by flow cytometry, expanded in culture, harvested, and suspended in Matrigel (BD Bioscience) before orthotopic transplantation of 1 × 10^6^ cells into the cortices of CD-1 nu/nu recipient mice, as previously described^8^. Animals were examined daily for symptoms of sickness (doming of the head, ataxia and reduced activity). Moribund mice were humanely sacrificed, tumor cells were harvested and RNA was extracted for further analysis. Tumors, brains, and spines of mice were reviewed by histopathology to evaluate whether the tumors were G3 MBs with large cell/anaplastic and metastatic features.

### Quantitative real-time PCR

Total RNA was extracted from tumor cells using Trizol (Thermo Fisher Scientific) and reverse transcribed into cDNA using the Multiscribe reverse transcription reagents according to the manufacturer’s protocol (Applied Biosystems). cDNA amplification was performed with an ABI 7900 Real-Time PCR system and FAM-Labeled Taqman primer/probes specific to the genes of interest (Applied Biosystems). Threshold cycle (CT) values from triplicate measurements were averaged and normalized to those obtained from the internal control gene, GAPDH. Relative gene expression was determined by the 2^−ΔΔCT^ method^49^. Data are expressed as the mean ± SEM. Statistical analyses were performed in GraphPad Prism Software v. 6.0. Statistical significance was determined by a Student’s T-test as indicated in the Figure legends.

### Affymetrix microarray analysis

RNA from tumor cells was subjected to hybridization using Affymetrix Mouse Gene 2.1 ST Array. The data were normalized and log2-transformed using RMA as implemented in Partek Genomic Suite 6.6 (St Louis, MO)^50^. The resulting matrix was joined to a RMA matrix of previously published mouse models from GSE65888 (arrayed on Affymetrix Mouse Genome 430 2.0 Arrays)^11^. The highest expressing probeset for each gene was used and the two array datasets were joined by gene symbol in STATA MP/14.2 yielding 17870 genes. Next using Partek 6.6, the data was corrected for the chip effect and visualized by PCA. Select genes of interest were after z-score normalized, hierarchically clustered, and presented in a “heat map” format.

### ChIP-Seq and data analysis

ChIP-Seq was performed exactly as described previously^8^. Chromatin was subjected to ChIP using rabbit polyclonal antibody directed against dCas9 (Clontech, #632607). To validate the dCas9 binding on their target sites, a primer pair spanning the target sites (M5) was used. PCR was performed using immunoprecipitated DNA and whole cell extract DNA as a control input. DNA was purified using phenol:chloroform and sequenced^51^ on the Illumina HiSeq platform; high-quality reads were aligned to the reference mouse genome (mm9) to visualize the sgRNA-mediated dCas9-DNA binding sites.

We used BWA (version 0.5.9-r26-dev, default parameter) to align the reads to the mouse (UCSC mm9) genome and then marked duplicated reads with Picard (version 1.65), with only non-duplicated reads kept by Samtools (parameter “−q 1 −F 1024” version 0.1.18). To control the quality of the data and estimate the fragment size, the non-duplicated version of SPP (version 1.11) was used to draw cross-correlation with support of R (version 2.14.0)^52^. Upon manually inspecting the cross-correlation plot generated by SPP, the best fragment size estimates (the smallest fragment size estimated by SPP in all of our cases) were used to extend each read and to generate bigwig files to view on the Integrative Genomics Viewer (IGV) (version 2.3.47). We scaled the bigwig files to 15 M reads so that the heights of peaks between samples are roughly comparable. To find the off-targets, MACS2 (version 2.0.10.20131216) was used to call peaks (−nomodel with fragment size estimated above) and low mapability region (50% of the peak mapability from wgEncodeCrgMapabilityAlign50mer < 0.5) were excluded. PatMaN was used to match the sequences summarized with extended reads counts by bedtools (v2.24.0)^53^.

### ATAC-Seq

ATAC-Seq was performed with 200,000 mouse ES cells and 75,000 MEF cells as described^54^. In brief, after preparation of nuclei, the nuclei were mixed with transposase for the digestion reaction. DNA was purified, amplified by PCR, and sequenced on a Hi-Seq 4000. Adapters were trimmed from total reads, which were then mapped to the mm9 mouse genome version. Mitochondrial DNA reads and duplicates were removed. Bigwig files were finally visualized using IGV.

### Additional statistical analyses

The Kaplan-Meier method was used to generate mouse survival curves. Statistical analyses were performed in the GraphPad Prism software version 6.0. P-values were calculated by an unpaired two-tailed t-test from three independent experiments.

### Data access

Accession numbers for the microarray and ChIP-Seq data reported in this paper are GEO: GSE102037 and GSE102096.

## Electronic supplementary material

Supplementary information
