# Supplementary material for: Mouse medulloblastoma driven by CRISPR activation of cellular Myc
Source: Sci Rep. 2018 Jun 7;8:8733. doi: 10.1038/s41598-018-24956-1 (PMC5992137; doi:10.1038/s41598-018-24956-1)
Supplement: Supplementary file 1 — Supplementary information [file 41598_2018_24956_MOESM1_ESM.pdf]

Supplementary Information

Mouse medulloblastoma driven by CRISPR activation of cellular *Myc*

BaoHan T. Vo, Jin Ah Kwon, Chunliang Li, David Finkelstein, Beisi Xu, Brent A. Orr, Charles J. Sherr & Martine F. Roussel

Figure S1. Epigenetic profiles of the Myc gene in different mouse G3 tumors. ChIP-Seq data of H3K27ac marks in tumor cells from three mouse Retro-Myc G3 MBs (MB1, MB2 and MB3) cell lines and neurosphere cells (NS) in the open chromatin region of the mouse Myc promoter. ATAC-Seq of MEF and mES cells. The location of the Myc promoter region used for the design of sgRNAs is noted by the highlighted black box.

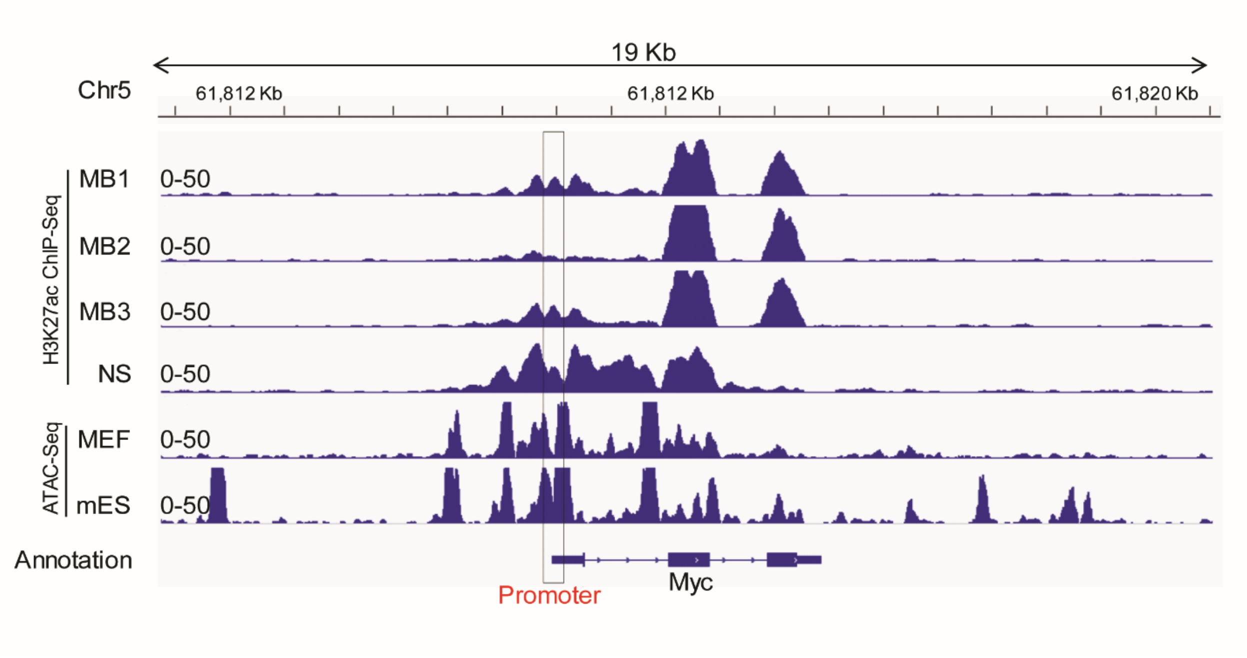

Figure S2. Quantitative RT-PCR of Myc expression in NIH3T3 cells. NIH3T3 cells transfected with U6, sgRNA-M5, M7 or M9 and primers that specifically bind to the 3'UTR **(a)** or the coding region **(b)** of the Myc promoter. Data are represented as the mean ± SEM.

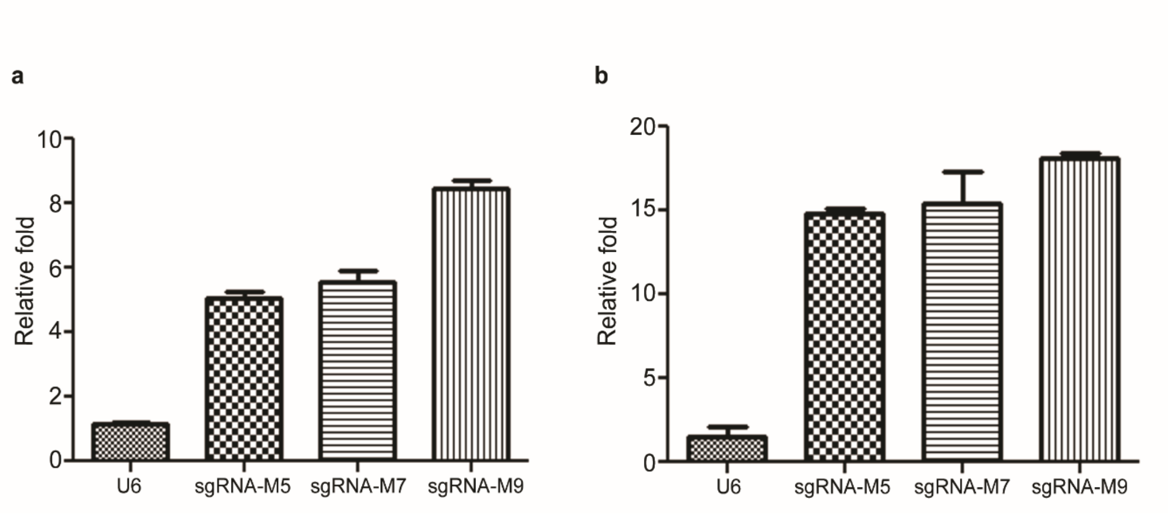

Figure S3. Characterization of the binding affinity of sgRNA-M5, 7 and 9 with dCas9-VP64 system, and indel frequency with wild type Cas9. ChIP-Seq data of H3K27ac marks in mouse neurosphere cells (NS), primary purified G3 tumor cells (G3 tumors), tumor cells from three Retro-Myc G3 MBs (MB1, MB2 and MB3) cell lines, and *Trp53*-null NS infected with lentiviruses expressing dCas9 and sgRNA-M5, 7, or 9. Top three off-target sites are located in genes *Gprc5c*, *Cystm1* and *Smyd5* identified by dCas9 ChIP-Seq in sgRNA-M7.

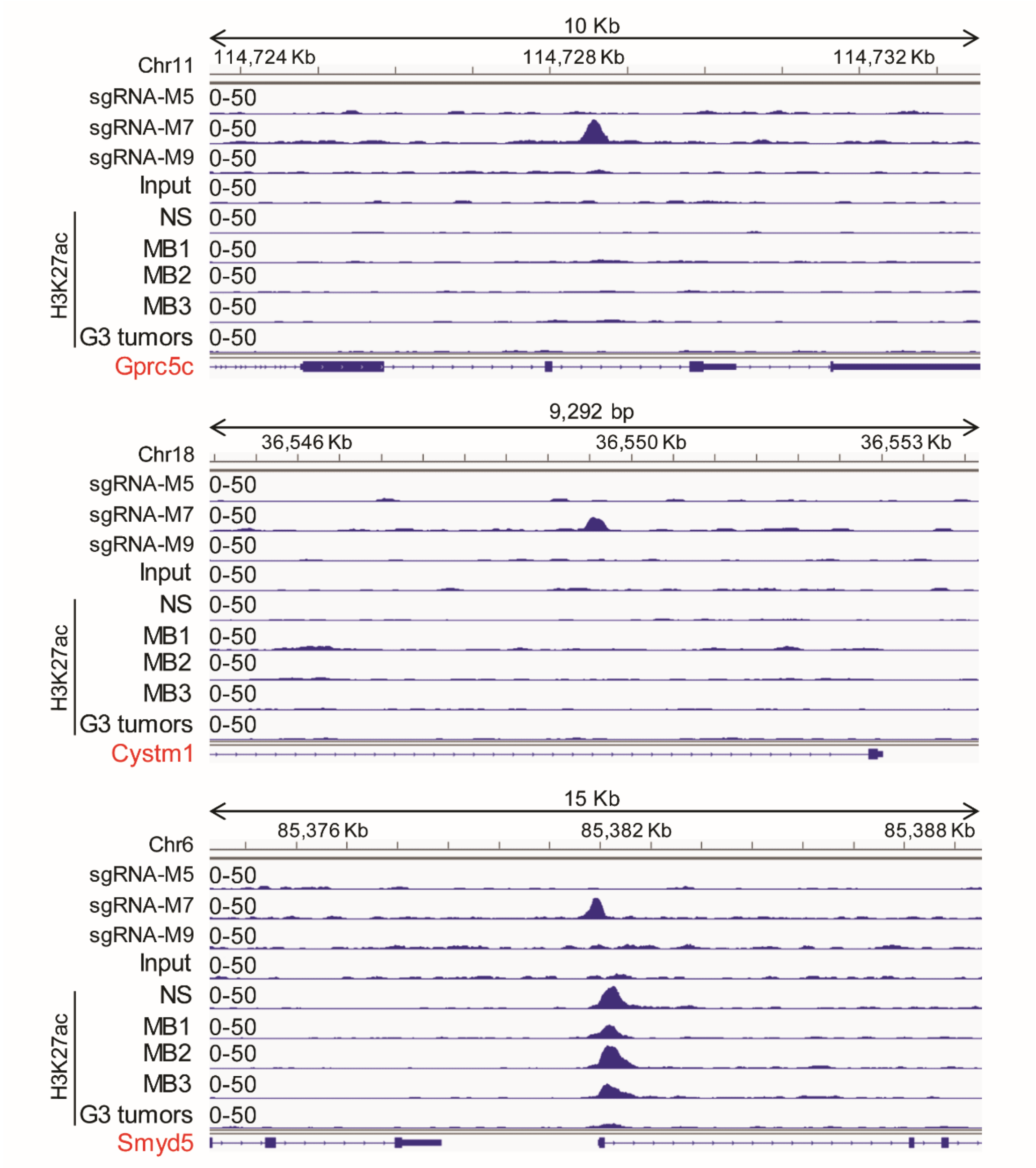

Figure S4. Full-length scan of immunoblots. Correspond to data presented in cropped Figure 1e.

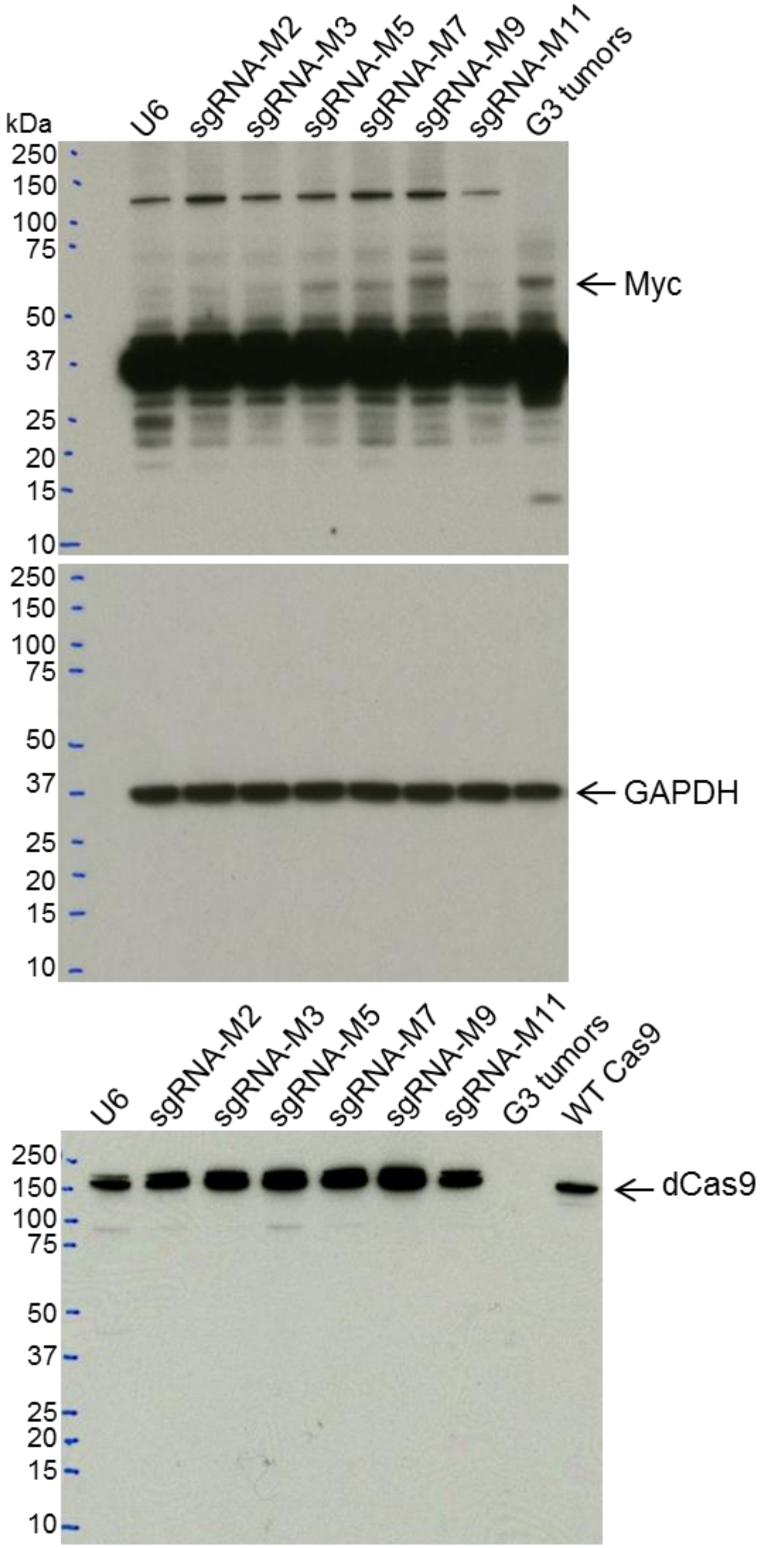

Figure S5. Full-length scan of immunoblots. Correspond to data presented in cropped Figure 2a.

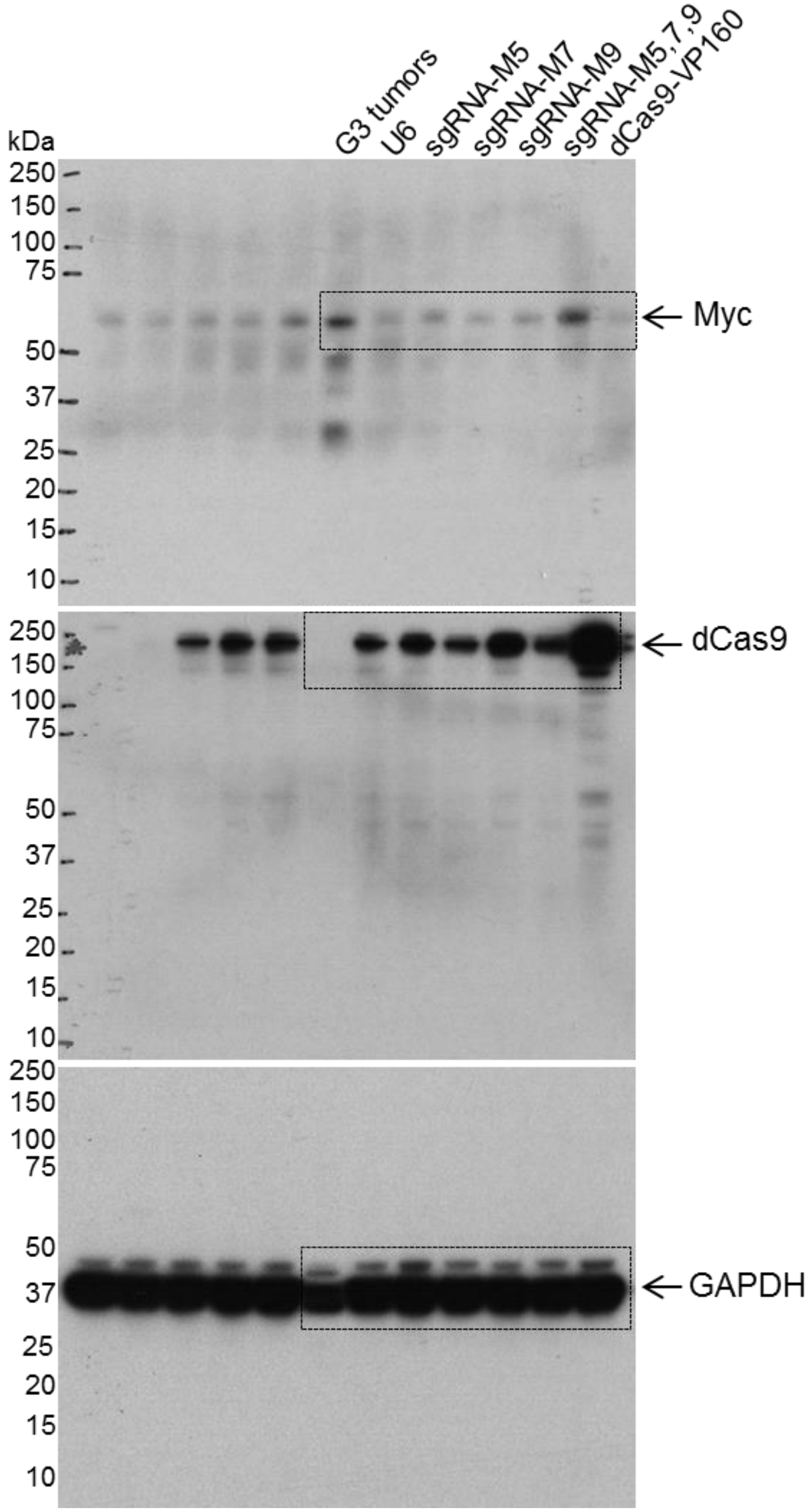

Figure S6. Full-length scan of agarose gel. Correspond to data presented in Figure 4a.

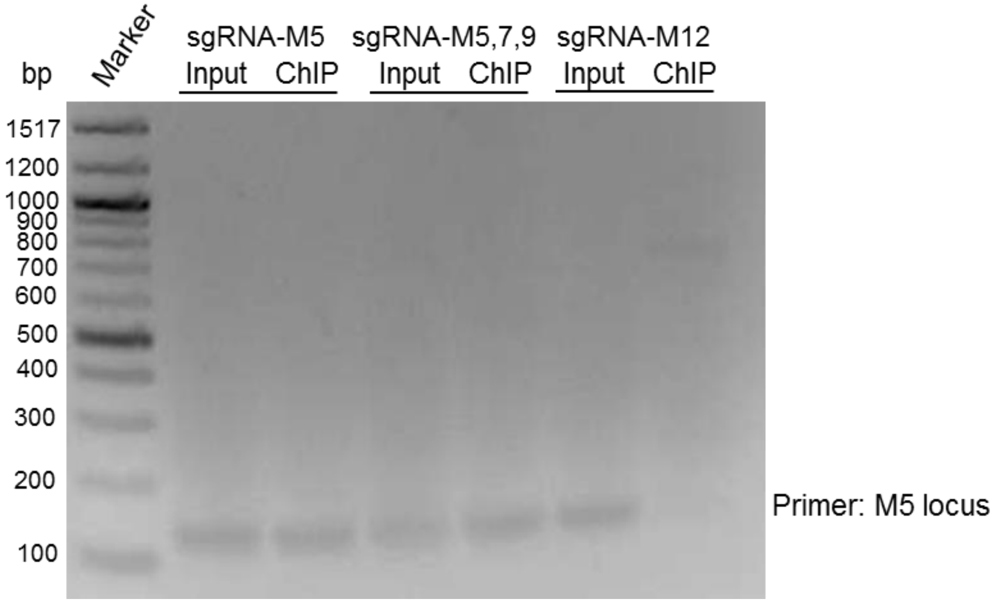

Figure S7. Full-length scan of immunoblots. Correspond to data presented in Figure 5a.

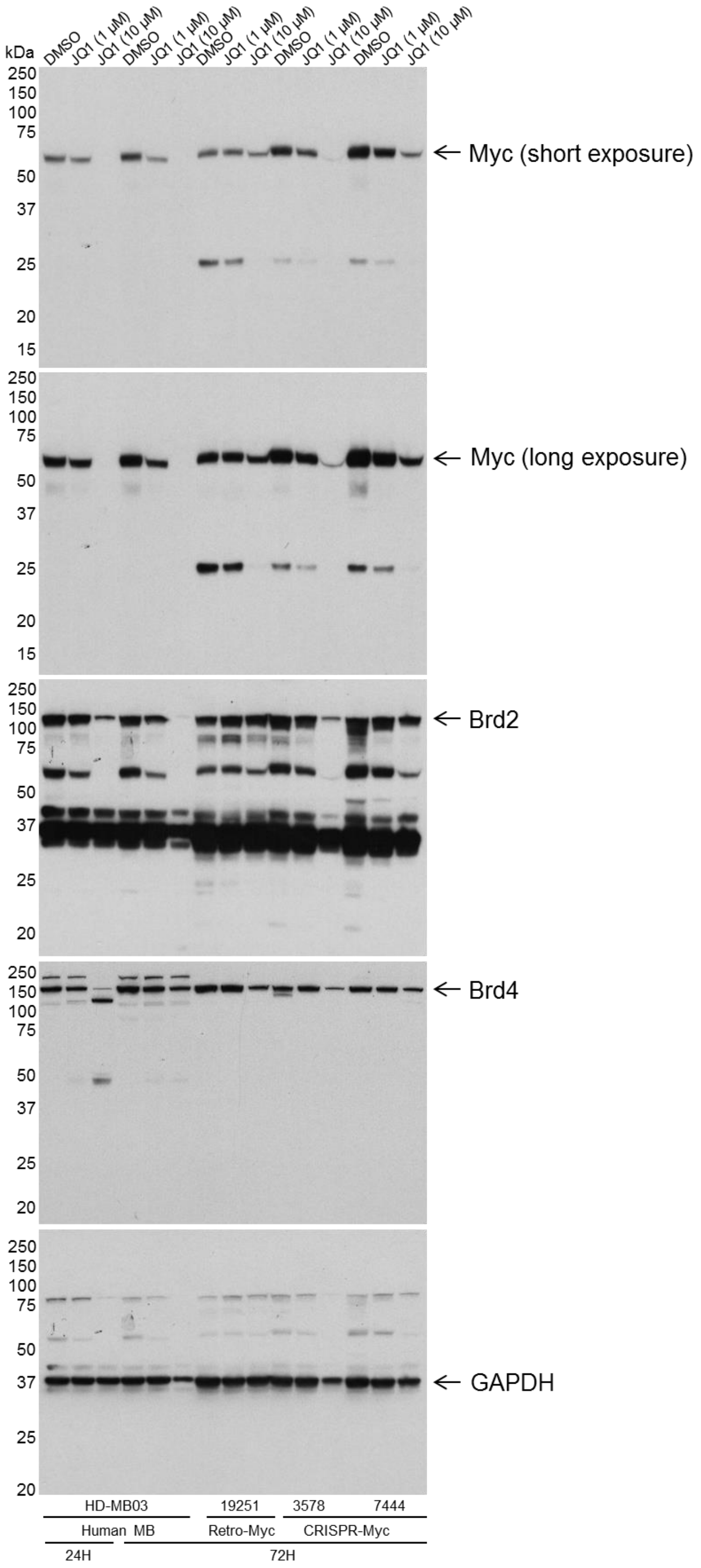

Supplementary Table S1. Oligomers used to generate target-specific sgRNA vectors.

Name	Sequence 5' to 3'
sgRNA-M2	GCTGATGTTGGGTCAGTCGC
sgRNA-M3	TCTTAGGTCGCCCCGGAGCG
sgRNA-M5	GGCAGGGCTTCGCCGACGCT
sgRNA-M7	AAATAGAGAGAGGTGGGGAA
sgRNA-M9	ACACCCCGGAGCCGGAGTAC
sgRNA-M11	GGTTGTTCGGGCGCAGCGCT
TALE-TF-1	TCAGCTCCCCTCCTGCCTCC
TALE-TF-2	TGAAGGGCAGGGCTTCGCCG
TALE-TF-3	TTCTCTGGCTAATCCCCGCC
TALE-TF-4	TAGAGAGAGGTGGGGAAGGG
TALE-TF-8	TACACCCCGGAGCCGGAGTA
